# A novel reconstruction method combining multi-detector SPECT with an elliptical orbit and computer tomography for cardiac imaging

**DOI:** 10.1038/s41598-023-42163-5

**Published:** 2023-09-11

**Authors:** Jinhua Sheng, Pu Huang, Rougang Zhou, Zhongjin Li, Xiaofan Yang, Jialei Wang

**Affiliations:** 1 School of Computer Science and Technology, Hangzhou Dianzi University, Hangzhou, 310018 Zhejiang China; 2 Key Laboratory of Intelligent Image Analysis for Sensory and Cognitive Health, Ministry of Industry and Information Technology of China, Hangzhou, 310018 Zhejiang China; 3 College of Mechanical Engineering, Hangzhou Dianzi University, Hangzhou, 310018 Zhejiang China; 4 Mstar Technologies Inc, Hangzhou, 310018 Zhejiang China

**Keywords:** X-ray tomography, Electrical and electronic engineering

## Abstract

The myocardial single photon emission computed tomography (SPECT) is a good study due to its clinical significance in the diagnosis of myocardial disease and the requirement for improving image quality. However, SPECT imaging faces challenges related to low spatial resolution and significant statistical noise, which concerns patient radiation safety. In this paper, a novel reconstruction system combining multi-detector elliptical SPECT (ME-SPECT) and computer tomography (CT) is proposed to enhance spatial resolution and sensitivity. The hybrid imaging system utilizes a slit-slat collimator and elliptical orbit to improve sensitivity and signal-to-noise ratio (SNR), obtains accurate attenuation mapping matrices, and requires prior information from integrated CT. Collimator parameters are corrected based on CT reconstruction results. The SPECT imaging system employs an iterative reconstruction algorithm that utilizes prior knowledge. An iterative reconstruction algorithm based on prior knowledge is applied to the SPECT imaging system, and a method for prioritizing the reconstruction of regions of interest (ROI) is introduced to deal with severely truncated data from ME-SPECT. Simulation results show that the proposed method can significantly improve the system's spatial resolution, SNR, and image fidelity. The proposed method can effectively suppress distortion and artifacts with the higher spatial resolution ordered subsets expectation maximization (OSEM); slit-slat collimation.

## Introduction

Nuclear cardiology is an essential component of modern heart disease management, as it allows physicians to gain insight into the structural and functional information of the heart^1^. Cardiac Single Photon Emission Computed Tomography (SPECT) utilizes two-dimensional projections of gamma photons emitted by an injected radiopharmaceutical to assess the radioactive tracer uptake in the patient's tissue. This technique can reveal areas of low tracer uptake in the heart muscle, which may indicate conditions such as coronary artery disease that impede blood flow. Many SPECT systems can be combined with Computed Tomography (CT) imaging, allowing for getting body structure information and compensating for the attenuation of emitted gamma photons^2^. Unlike Positron Emission Tomography (PET), SPECT is often more cost-effective and readily available^3^. Due to its versatility and utility, SPECT has a wide range of clinical applications.

Over the past few decades, the development of SPECT has evolved along two main trajectories: miniaturization for wider clinical use in office-size settings and specialization for larger systems. However, current cardiac SPECT systems have limitations, such as low photoelectric signal conversion rate and low sensitivity. As the photons detected by the detectors come from radioactive materials injected into the body, there is a maximum for safe injection doses to prevent damage to the human body, which results in a low SNR. Furthermore, many small SPECT systems do not integrate CT for attenuation compensation (AC), which diminishes image reconstruction quantification. Additionally, the angle and orbit of the detectors can limit the sampling efficiency of SPECT.

To address these issues, many solutions have been proposed. Dynamics (Haifa, Israel) proposed the D-SPECT. Using multi-detector parallel sampling and semiconductor detector and list mode solves the problem of low sampling efficiency and improves the system time resolution. Digirad, Inc. (Poway, CA) developed the Cardius 3 XPO (C 3 XPO), which uses three cameras and an intelligent rotating chair, providing enough sampling angles and faster sampling speed^4, 5^. However, the above designs still have disadvantages. The former needs more angles for projection acquisition, while the latter does not solve the problem of low photoelectric conversion efficiency. In addition, both models have successfully been manufactured and sold, but neither has effectively addressed the image reconstruction quality defects caused by the lack of integrated CT.

In SPECT reconstruction, merging high-resolution CT data enhances resolution and lesion detection, as seen in SPECT/CT's utility for coronary artery calcium scoring^6^. Our goal is to develop a high-quality SPECT/CT system to effectively integrate CT, and to design imaging methods based on the characteristics of system parallel sampling. To attain this objective, we plan to incorporate the information obtained from CT with the parameters adjustment of the slit-slat collimator employed, ensuring that the Field of View (FOV) of multiple detectors remains aligned with the location of the heart. Furthermore, Sampling along non-circular or elliptical orbits is established, prompting new sampling logic. Therefore, we attempt to present a multi-detector hybrid myocardial imaging SPECT/CT system that incorporates elliptical orbits, thereby achieving a balance between spatial resolution and photon sensitivity. And implemented within the framework of the proposed system's sampling logic, our algorithm harnesses the reciprocal interaction between ROI and non-ROI as prior knowledge, thereby achieving the reconstruction of high-quality images.

## Materials and methods

The proposed method was implemented in MATLAB. All methods were carried out in accordance with relevant guidelines and regulations. All experimental protocols were approved by the institutional review board (IRB) at Hangzhou Dianzi University (IRB-2020002).

### Description of the multi-detector elliptical SPECT System

Multi-detector elliptical SPECT (ME-SPECT) is proposed to be an adaptive SPECT/CT platform that configures each collimator's parameters based on the results of the CT reconstruction. The SPECT component includes eight independent detectors and slit-slat collimators. In our conception, each SPECT detector continuously acquires photons by aligning with the ROI based on the CT reconstruction results. Each slit-slat collimator corresponds to a detector and samples the human body rotation along the set orbits, adjusting its parameters based on the current position and CT information to achieve a balance between spatial resolution and photon sensitivity. We have also proposed a new reconstruction algorithm for ME-SPECT.

#### ME-SPECT Collimators and Detectors

**Figure 1:**
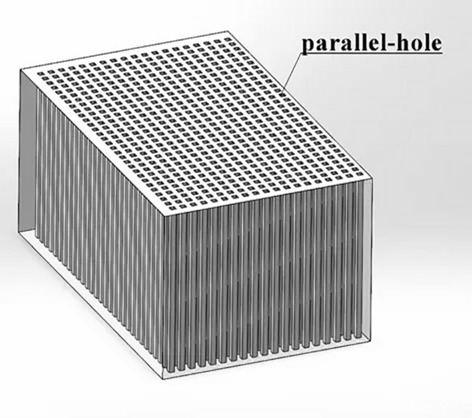
Parallel-hole collimator.

Choosing the right collimators and detectors is crucial for high-quality reconstructed images in SPECT. Collimators are made of high-density materials and control the direction of photons received. Traditional SPECT uses parallel-hole collimators with a well-controlled FOV and sensitivity that doesn't decrease with distance^7^, as shown in Fig. 1.

**Figure 2:**
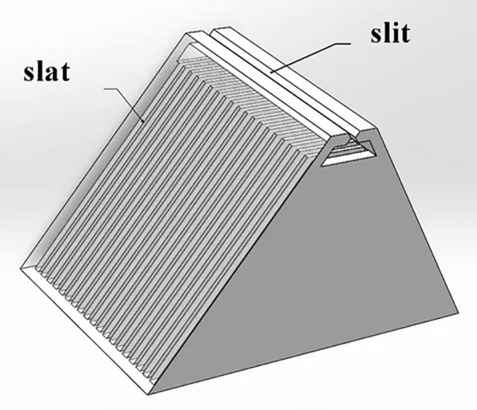
Slit-slat collimator.

On the other hand, ME-SPECT utilizes the slit-slat collimator, which is a hybrid of parallel-hole and pinhole collimators. The slit-slat collimator has a long knife edge in the transaxial direction and parallel slats in the axial direction, as shown in Fig. 2, providing high-resolution imaging in the transaxial plane for small to medium-sized targets. Therefore, ME-SPECT chose the slit-slat collimator to achieve superior performance in the transaxial plane for small to medium-sized targets.

In SPECT imaging, the gamma-ray detector plays a crucial role in detecting and converting gamma photons into electrical signals. Conventional SPECT utilizes scintillation detectors such as NaI, which have limitations in photon conversion efficiency and intrinsic spatial resolution, resulting in low-quality images^8, 9^. Meanwhile, ME-SPECT employs semiconductor detectors like the CZT detector, which functions as a direct conversion device and offers better energy resolution and intrinsic spatial resolution of 2.4mm^10^. CZT detectors have the advantage of room-temperature operation, intermediate mass, and low scattering, making them suitable for detecting small lesions. Although germanium detectors have the highest energy resolution, their low-temperature operation requirement makes them less feasible for ME-SPECT^8^. By utilizing CZT detectors, ME-SPECT can achieve high-quality reconstructed images with high spatial resolution, providing a useful diagnostic tool for detecting small lesions. Conventional SPECT's utilization of scintillation detectors with PMTs and more significant spatial limitations is incompatible with the design criteria for myocardial SPECT.

#### Elliptical Orbit SPECT Sampling System

**Figure 3:**
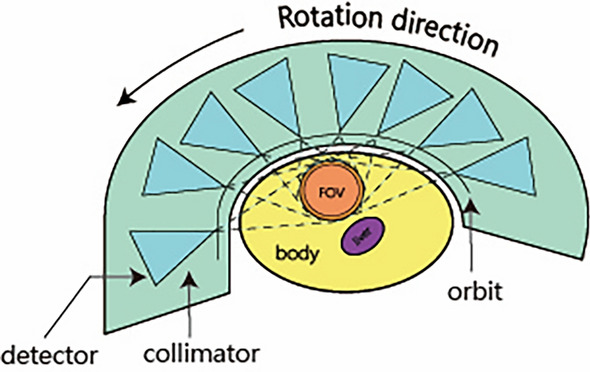
Collection of projection data by the SPECT detector along the orbit.

In the preceding discussion, we have outlined the hardware components involved in ME-SPECT. Based on these components, we have designed a sampling system for the SPECT. Previous studies have demonstrated that the accuracy of sampling and the spatial resolution of SPECT detectors are influenced by the distance between the detection equipment and the ROI due to factors such as the geometry of the collimator^11^. Therefore, to enhance the precision and accuracy of our measurements, we have opted for an elliptical sampling orbit closer to the human body, as shown in Fig. 3.

In the model shown in Fig. 3, each detector samples in a counterclockwise direction along an elliptical orbit with a long-axis radius of 240 mm and a short-axis radius of 168 mm. The sampling starting angle is 15°, and the ending angle is 195°, providing a total coverage angle of 180°. During the sampling process, each detector rotates 21°, rotating 3° each time, and eight samples are collected. Based on the multi-detectors’ design, eight detectors can sample simultaneously, resulting in 64 (8 × 8) sets of projections. It's important to highlight that each projection data originates from photons traveling through collimators to reach the detector array. Therefore, this model will outperform traditional dual-head SPECT regarding sampling time.

**Figure 4:**
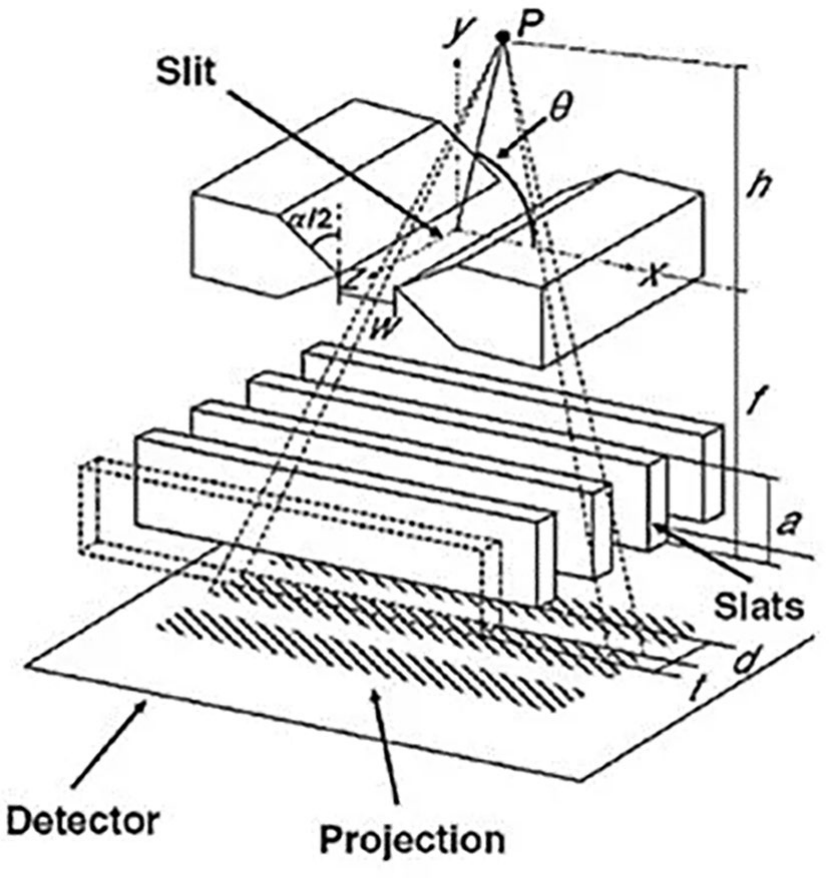
Perspective conceptual drawing of slit–slat collimator^11^.

As previously mentioned, the slit-slat collimator was chosen due to the size of the ROI and the goal of achieving high spatial resolution. The conceptual diagram of the slit-slat collimator^11^ is shown in Fig. 4.

The diagram depicts the *x*, *y*, and *z* axes, with *P* representing the emission source, *θ* representing the incident angle, *h* representing the distance from the emission source to the slit, *f* representing the focal length of the collimator, *w* representing the width of the slit, *t* representing the thickness of the slat, *d* representing the gap between the slats, and a representing the length of the slats.

In terms of horizontal axis resolution, the spatial resolution of the slit-slat collimator is equal to that of a pinhole collimator^11^:

1 $$\begin{array}{c}{R}_{0}\left(transaxial\right)={R}_{0}\left(pinhole\right)=\sqrt{{R}_{g}^{2}\left(pinhole\right)+{\left(\frac{h}{f}{R}_{i}\right)}^{2}}\\ =\sqrt{{w}^{2}{\left(\frac{h+f}{h}\right)}^{2}+{\left(\frac{h}{f}{R}_{i}\right)}^{2}}\end{array}$$

Additionally, the longitudinal spatial resolution is maintained to be equivalent to that of parallel-hole collimators^11^:

2 $$\begin{array}{c}{R}_{0}\left(axial\right)={R}_{0}\left(parallel\right)= \sqrt{{R}_{g}^{2}\left(parallelbeam\right)+{R}_{i}^{2}}\\ =\sqrt{{d}^{2}{\left(\frac{f+h}{a}\right)}^{2}+{R}_{i}^{2}}\end{array}$$

where *R* represents the system spatial resolution, ${R}_{g}$ represents the collimator spatial resolution, and *R*_*i*_ represents the intrinsic spatial resolution of the detector. Once the parameters of the collimator are determined based on the spatial information, the sensitivity of the collimator can be calculated using Eq. (3)^12^:

3 $$\begin{array}{c}g\left(\mathrm{slit}-\mathrm{slat}\right)=g\left(\mathrm{pinhole}|\mathrm{parallel}\right)\\ =\sqrt{g\left(pinhole\right)\times g\left(parallel\right)} \\ =\sqrt{\frac{{w}^{2}}{4\pi {h}^{2}}{sin}^{3}\left(\theta \right)\times \frac{{d}^{4}}{4\pi {a}^{2}{\left(d+t\right)}^{2}}{sin}^{3}\left(\theta \right)}\\ =\frac{w{d}^{2}}{4\pi ah\left(d+t\right)}{sin}^{3}\left(\theta \right)\end{array}$$

Equations (1) and (2) independently validate the resolutions of pinhole and parallel-hole collimators in the transverse and axial directions, respectively. Taking into account the influence of material penetration, Accorsi and Metzler^12^ derived a resolution-effective diameter dependent on *θ*, which accounts for penetration for a double-knife edge pinhole collimator. Thus, the effective diameter in the transverse direction for the slit-slat collimator can be determined as follows:

4 $$\begin{array}{c}{w}_{re}=w+\frac{\mathrm{ln}2}{\mu }\mathrm{sin}\theta \mathrm{cos}\frac{\alpha }{2}{\mathrm{tan}}^{2}\frac{\alpha }{2}-{\mathrm{cos}}^{2}\theta \end{array}$$

As the above equation demonstrates, increasing the collimation slit gap hurts the transverse spatial resolution but positively impacts sensitivity. In the design of collimator parameters, we need to consider the spatial resolution, sensitivity, and spatial constraints. The distance from the slit to the detector significantly impacts the transverse spatial resolution and sensitivity. However, a too-large *f* results in a reduction in the number of detectors for parallel sampling. Previous studies proposed a design where each collimator parameter differs^13, 14^. We believe that this design lacks universal applicability to different human bodies. After considering all factors, we adopted eight detectors for parallel sampling, with each detector having the same parameters except for the slit angle, as shown in Table 1.

**Table 1:** Collimator configuration parameters.

w(mm)	d(mm)	t(mm)	f(mm)	a (mm)
2	3.5	0.1	110	102

#### Combination of ME-SPECT and CT

In the context of a multi-detector SPECT/CT system, the information provided by the CT is critical in AC, ROI localization, and incorporating prior constraints. Integrating CT into SPECT in the same device significantly reduces interference.

**Figure 5:**
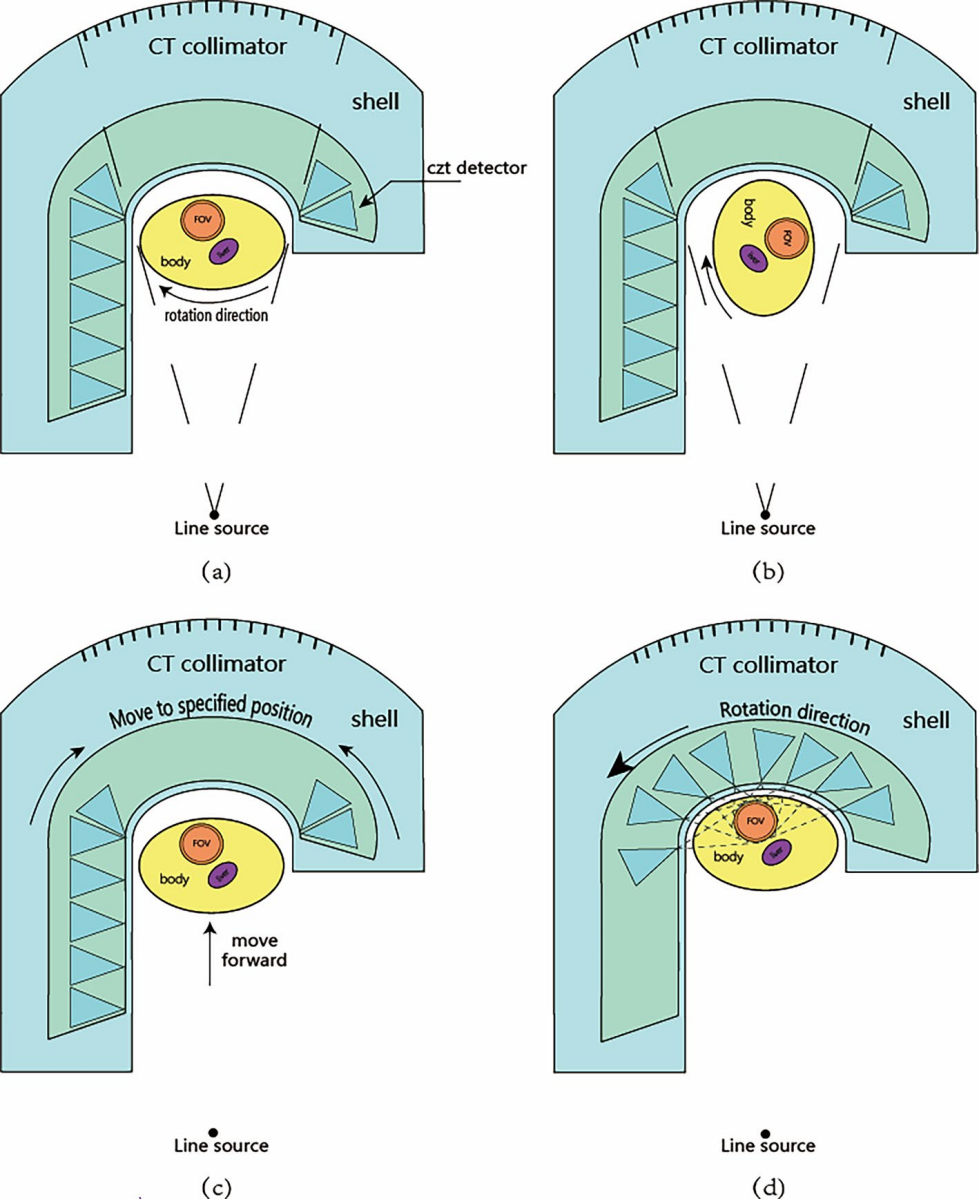
The SPECT/CT acquisition process of ME-SPECT.

However, incorporating CT into small myocardial SPECT requires consideration of spatial limitations. The CT radiation source is external, making it challenging to rotate the source around the body in an office-sized environment. The proposed ME-SPECT implements a strategy of keeping the radiation source and detector stationary while a smart chair rotates the body for CT sampling, as shown in Fig. 5.

Figure 5 shows a fan beam that covers the entire human body as a point source of light, with the fan CT detector placed behind the SPECT orbit. When the CT is sampled, the SPECT detector moves along the orbit to the side. The X-ray source is then turned on, allowing the X-ray to pass through the unobstructed SPECT orbit and irradiate the CT detector from various angles as the intelligent chair rotates smoothly with the human body, as shown in Fig. 5a,b. After CT sampling, the X-ray source is turned off, and the adjacent SPECT detector returns to its starting position while the intelligent chair moves toward the direction closer to the SPECT, as shown in Fig. 5c. When the detector and human body are in place, the SPECT detector begins to rotate counterclockwise along the orbit. At each position, the detector adjusts the slit width and sampling angle based on the CT results, ensuring that the detector is always aligned with the ROI and rotates counterclockwise along the orbit until the sampling is completed.

Integration of CT data with SPECT imaging can improve the reconstruction of SPECT images, which are often limited by attenuation, scattering, and spatial resolution. Patient attenuation correction using CT images, which can provide high-quality information about tissue attenuation, is a valuable source for SPECT imaging. SPECT/CT imaging has lower noise levels and improved image quality compared to separate SPECT and CT acquisitions. In D-SPECT, the detector can localize the ROI through a pre-scanning mechanism, which is simple and efficient given the fixed position of the detector along the orbit. However, the proposed ME-SPECT system requires detector rotation to obtain a comprehensive projection data set. Incorporating an integrated CT scan dramatically improves the accuracy of ROI localization. It enhances the system's versatility by allowing adjustment of the FOV angle of each collimator based on the relative position between the ROI and the detector.

### SPECT imaging algorithm

In the emission tomography field, various algorithms have been developed for image reconstruction. Reconstruction algorithms can be broadly classified into two categories: analytical algorithms and iterative algorithms. Analytical algorithms produce images using inverse solution formulas and frequency domain density function relationships. The most commonly used algorithm in this category is the Filtered Back Projection (FBP) algorithm, which can correct for weighted Fourier space and produce high-quality images at a quick pace^13–16^. However, FBP has some limitations, such as the need for re-derivation when adding constraints and suboptimal image quality in truncated regions.

In contrast, iterative algorithms are based on algebraic methods and can simulate photon emission and detection processes, generate statistical noise models, and incorporate various constraints^17^. Despite their advantages, they require extensive computation, resulting in longer reconstruction times. In the proposed ME-SPECT model, FBP is used for CT component reconstruction. In contrast, iterative algorithms are used for SPECT imaging to capitalize on the information gained from the CT imaging component.

#### Formula and principle of the iterative algorithm

The data model is based on a Poisson statistical model, which assumes that the number of photons each detector receives from each projection ray follows a Poisson distribution. Many studies have also demonstrated that the random noise in SPECT is Poisson distributed^18, 19^. Thus, the actual number of photons received can be represented as:

5 $$\begin{array}{c}Y\sim Possion\left\{CX+R\right\}\end{array}$$

where $Y={[{y}_{1}, {y}_{2},\dots ,{y}_{M}]}^{T}$ is an $M\times 1$ dimensional vector of received projection data, with M representing the total number of received projections. *C* is a $M\times N$ matrix that represents the system response matrix, with *N* representing the total number of pixels in the image. $X=[{x}_{1},{x}_{1},\dots ,{x}_{N}]$ is an $N\times 1$ dimensional vector representing the image vector. $R=[{r}_{1},{r}_{1},\dots ,{ r}_{M}]$ is a $M\times 1$ dimensional vector representing the noise, scatter, and other effects that impact the number of photons received, with ${r}_{i}$ representing the impact on the ${i}^{th}$ projection.

From Eq. (5), it is evident that iterative statistical reconstruction determines the image vector *X* based on the known projection value *Y* and the response matrix *C*. The response matrix *C* can be calculated based on the proposed ME-SPECT model orbit and the design of the slit-slat collimator at the corresponding sampling position.

The OSEM algorithm is a variant of the maximum-likelihood expectation–maximization (MLEM) algorithm, and it divides the projection data into several ordered subsets. During each iteration, one subset is selected for iteration. Therefore, compared to the MLEM algorithm, the OSEM algorithm has higher efficiency^20, 21^. This algorithm with a relaxation factor can be written as:

6 $${\widehat{x}}_{j}^{n+1}={\widehat{x}}_{j}^{n}+{\eta }_{k}\frac{{\widehat{x}}_{j}^{n}}{\sum_{i=1}^{M}{c}_{ij}}\sum_{i\in {S}_{t}\left(k\right)}^{M}{c}_{ij}(\frac{{y}_{i}}{\sum_{l=1}^{N}{c}_{il}{\widehat{x}}_{l}^{n}}-1)$$

In Eq. (6), the relaxation factor ${\eta }_{k}$ is used to regulate the step length in the iteration process to prevent the algorithm from converging slowly or oscillating. Sheng et al. proposed a method to adjust the size of the subset and the iteration step length during the iteration process to recover various frequency components in the early iteration process^22, 23^. When the size of the subset changes, the adjustment of the relaxation factor is also necessary. ${S}_{t}(k)$ represents the ${k}^{th}$ subset, and ${S}_{t}$ is the set of subsets after division.

#### System response matrix

Calculating the system response matrix requires a comprehensive understanding of the signal propagation process and the specific parameters of each collimator in the system. The geometry between each component also needs to be taken into account. Finally, an appropriate mathematical model is chosen based on the desired level of accuracy for solving the system response matrix.

There are several methods for solving the system response matrix, such as the layer projection method and the physical simulation method^24^. Siddon transform was adopted in this work due to its intuitiveness, efficiency, and high accuracy. Siddon transform ensures efficient computation when dealing with complex scenarios by dividing the propagation path into segments and calculating each segment discretely.

**Figure 6:**
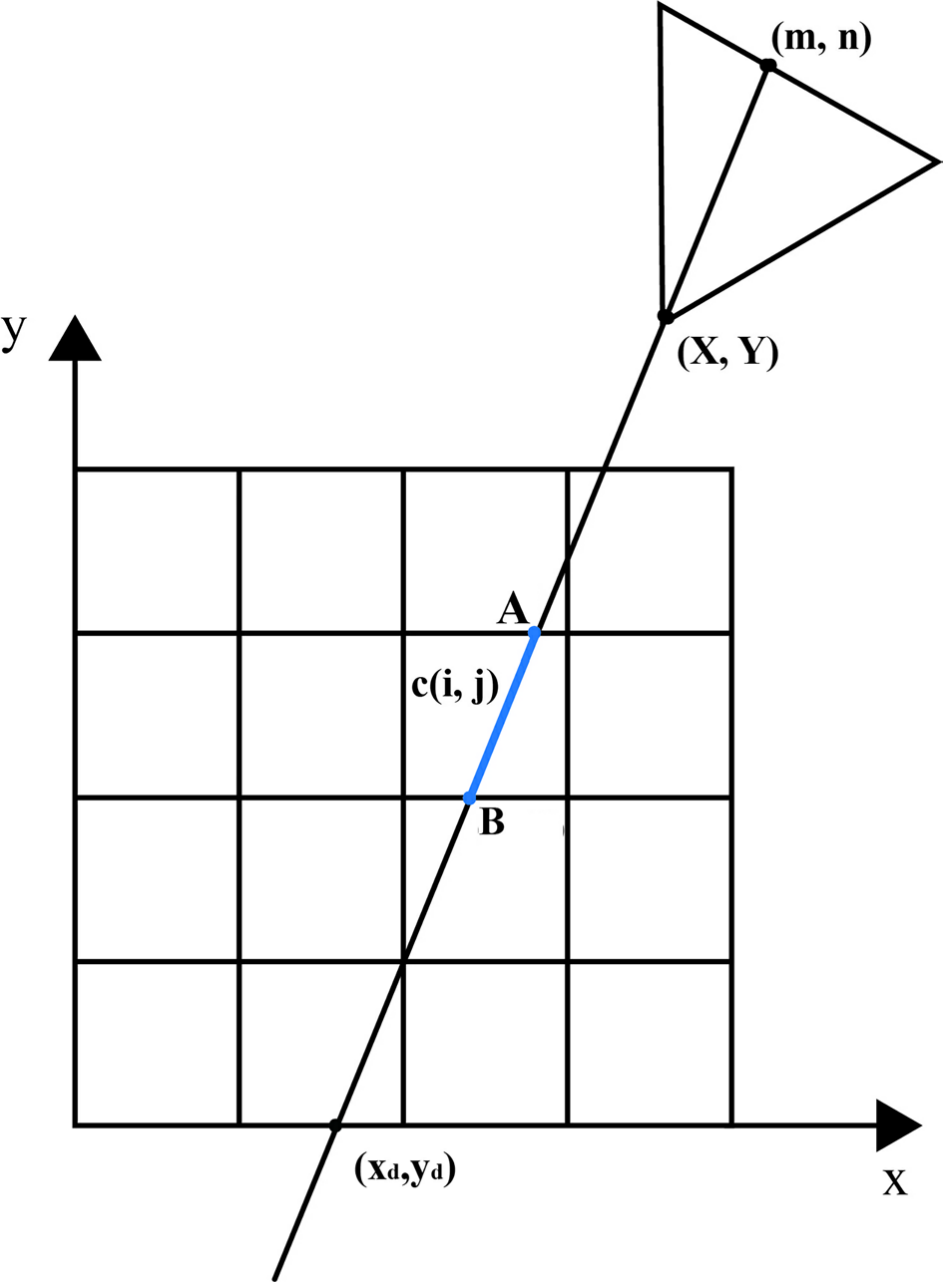
Computing system response matrix model using Siddon transformation**.**

The Siddon transform computational system response matrix is depicted in Fig. 6. In Fig. 6, the photon propagation path passes through the image matrix and collimator to reach the detector. Point *A* and Point *B* are the intersection points between the propagation path and the image, while $\left(X,Y\right)$ represents the entrance of the slit-slat collimator, and $({x}_{d},{ y}_{d})$ represents the detector coordinates receiving the photons. The transfer matrix C can be represented as:

7 $$\begin{array}{c}c\left(i,j\right)=abs\left(\frac{planes\left(i\right)-X}{X-{x}_{d}}-\frac{planes\left(j\right)-Y}{Y-{y}_{d}}\right)\times L\end{array}$$

In Eq. (7), $c\left(i,j\right)$ represents the length of the photon path within the ${i}^{th}$ row and ${j}^{th}$ pixel, while $planes(i)$ represents the boundary of $c\left(i,j\right)$ on the x-axis and $planes\left(j\right)$ represents the boundary on the y-axis. *L* represents the distance between $(X,Y)$ and $({x}_{d},{y}_{d})$.

#### Attenuation compensation

In the above formulation, AC’s effect must be considered while discussing image reconstruction. AC is a process used to correct for the reduction in the number of photons received due to absorption by the tissue.

There are many methods for performing decay compensation on images, such as the correction of the tomogram image after image reconstruction proposed by Chang et al.^25^. The reconstruction method with exponential correction during back projection was proposed by Tretika et al.^26^. In ME-SPECT, it is possible to use the attenuation mapping image obtained from the CT image to correct the system response matrix, as shown in the following figure:

8 $$\begin{array}{c}{a}_{ij}={c}_{ij}{e}^{-\sum_{k\in {L}_{ij}}{d}_{ik}\times {\mu }_{k}}\end{array}$$

where in Eq. (8), ${a}_{ij}$ represents the corrected system response matrix, and *L* represents the set of pixel boundaries that the path from photon *i* to detector *j* passes through. ${d}_{ik}$ is the distance from detector *i* to pixel *k*, and ${\mu }_{k}$ is the attenuation factor of pixel *k*. Thus, the algorithm in Eq. (9) can be adjusted as follows:

9 $${\widehat{x}}_{j}^{n+1}={\widehat{x}}_{j}^{n}+{\eta }_{k}\frac{{\widehat{x}}_{j}^{n}}{\sum_{i=1}^{M}{a}_{ij}}\sum_{i\in {S}_{t}\left(k\right)}^{M}{a}_{ij}(\frac{{y}_{i}}{\sum_{l=1}^{N}{a}_{il}{\widehat{x}}_{l}^{n}}-1)$$

#### ME-SPECT reconstruction process

In the ME-SPECT reconstruction, the CT part is reconstructed first, followed by the SPECT part. The CT part can be reconstructed using the FBP algorithm due to its sufficient angle coverage during data collection. However, in the SPECT part, the image is severely truncated due to the high sensitivity alignment of the ROI during data collection. Therefore, we need to add some constraints to complete the image reconstruction.

The CT information can be used to obtain the ROI's body contour and the location and size information. Adding prior knowledge of the body contour information during reconstruction can effectively improve the quality of the reconstructed image^27^. The specific operation sets the area outside the body contour in the initialized image to 0 and the area inside the body contour to the average body density.

In conjunction with adhering to the contours of the body, the reconstruction process benefits from the inclusion of both location and size data regarding the Region of Interest (ROI). Even with the incorporation of body contour constraints, addressing the significant truncation inherent in data sampling remains essential to facilitate the reconstruction of non-ROI regions. Neglecting to address the blurring of non-ROI elements can significantly compromise the overall quality of ROI reconstruction.

In the proposed ME-SPECT methodology, when the initial density of non-ROI approximates the average human density, maintaining the initial state of the non-ROI during iterative processes can yield an inferior ROI image. During this stage, the non-ROI appears smooth, while the ROI reveals the rudimentary form of the initial reconstruction outcomes. To counteract this, the initial body contour image and the subpar ROI image are amalgamated to serve as the starting point for iterative refinement.

Given the inherent smoothness of the non-ROI at the outset of iteration, the nascent ROI formation throughout the iterative process serves as a prior constraint. This impels subsequent iterations to progressively yield a diffuse image of regions like the liver. Our investigation identified that achieving a closer match between the weighted sum of non-ROI areas intersected by a photon-receiving detector at a specific angle and the actual image results in heightened accuracy of ROI reconstruction.

**Figure 7:**
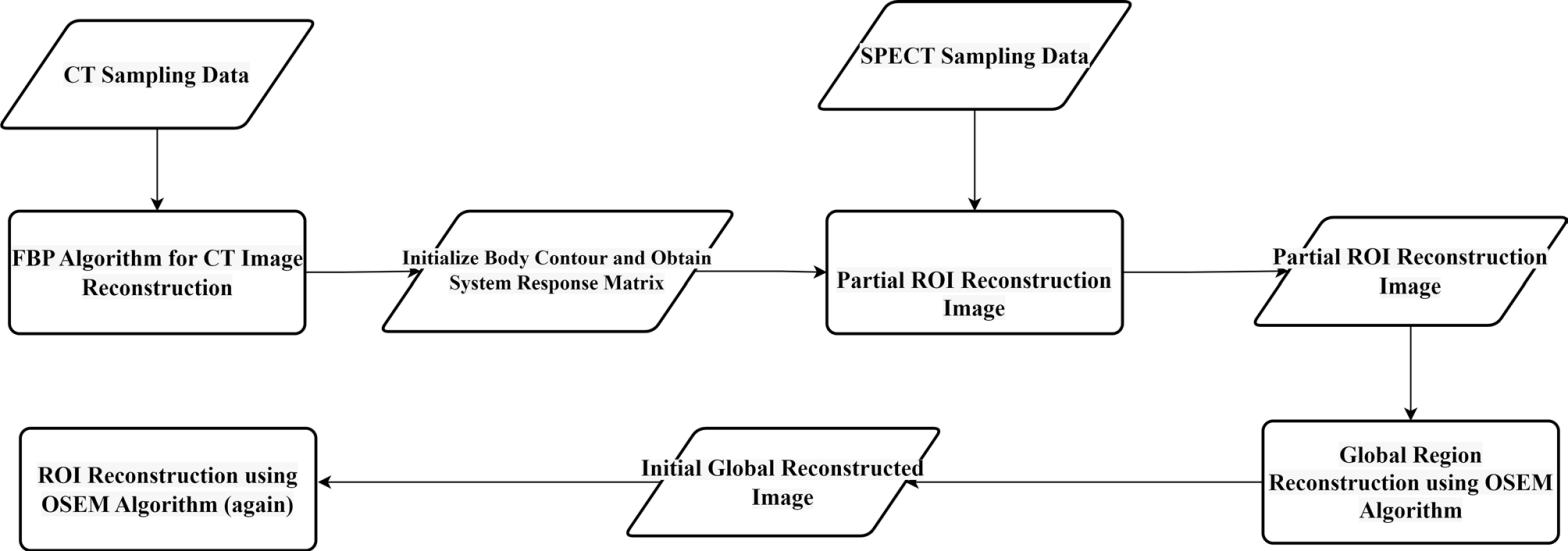
Workflow of the proposed SPECT reconstruction algorithm.

In contrast to conventional ROI-focused reconstruction algorithms, our approach distinctly segregates ROI and non-ROI for separate reconstruction, thereby enabling a mutual feedback loop between these two distinct regions. Simultaneously, this method adeptly mitigates artifacts stemming from portions of the ROI that remain unscanned by multiple detectors. In the SPECT reconstruction process, photons are assumed to be received as rays. As a result, there is a large amount of linear noise in the reconstructed image, and the median filter can effectively remove the related noise and improve the reconstruction quality. In addition, a non-negative constraint must be added during the reconstruction process. The algorithm's procedural diagram is illustrated in Fig. 7.

The specific algorithm is outlined as follows:
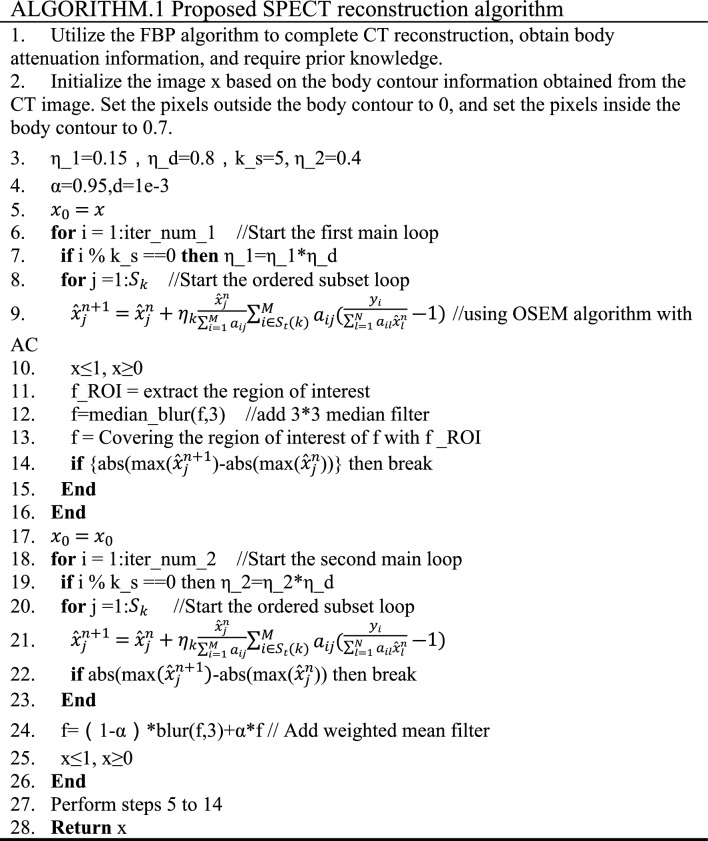


The symbol "//" is a notation used to denote a comment. The parameter defined in step 3 regulates the step size for gradient descent during both iterations. The parameters specified in step 4 include the filter weighting factor and the condition for ending the loop. Step 10 of the algorithm guarantees non-negativity and values less than 1 for the data. Steps 11–13 ensure that the initial reconstruction is confined within the ROI. Step 14 defines the conditions under which the loop will end prematurely. The outcome of the previous iteration is used as the starting image in step 17. In steps 18 to 26, the second reconstruction is carried out. As the non-ROI may be reconstructed in this iteration, a weighted median filter is applied for denoising purposes. The data is ensured to be non-negative and less than 1 after each iteration of all subsets. Step 27 is the addition of a median filter to remove noise based on the reconstruction result from the previous iteration, to improve the reconstruction quality in the ROI.

## Simulation

**Figure 8:**
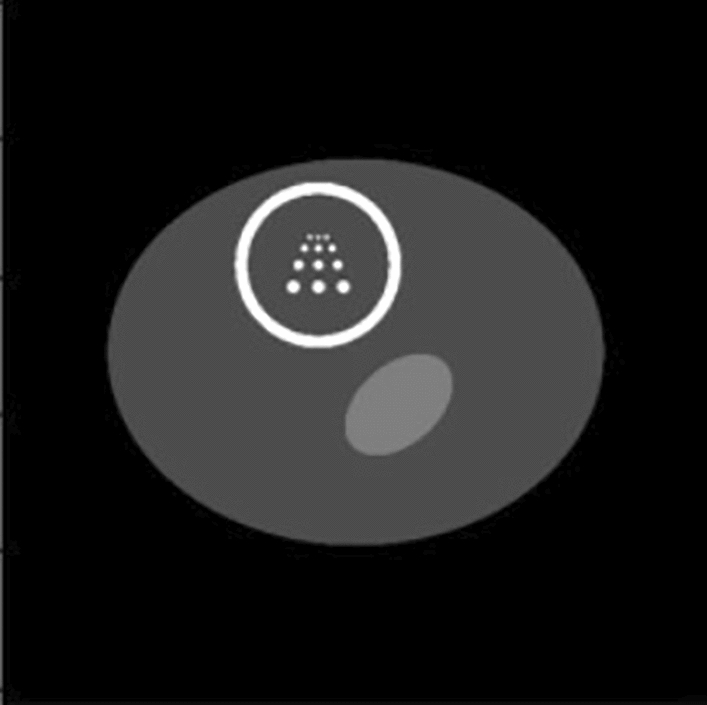
Cardiac phantom.

In this article, we present a phantom for evaluating the quality of myocardial SPECT/CT reconstruction. The data used for reconstruction will be computed from the phantom using a collimator that conforms to the designated parameters and is positioned along the sampling trajectories illustrated in Fig. 7, as outlined by the statistical methodology in Eq. (6). Since the main objective of this simulation is to evaluate the accuracy of the reconstructed truncated projection region, the noise was not added during the generation of the phantom. The design of this phantom was referenced the Chinese Visible Human Project dataset^28^. The myocardial phantom is shown in Fig. 8. There isn’t any experiments on the use of human tissue samples involved in this study.

As depicted in Fig. 8, the phantom depicts a cross-section of a human body encompassing the heart. The outer ellipse symbolizes the human body, with a density of 30%. With a long axis radius of 200 mm and a short axis radius of 140 mm, the ellipse's dimensions reflect the overall proportions of the human body. The smaller ellipse within symbolizes the liver, with a density of 50%. Its long-axis radius is 45 mm, and its short-axis radius is 29 mm, with a rotation angle of 41°. The circular shape in the upper left corner of the figure represents the heart, which is the main reconstruction target. The circle has a radius of 60 mm, with a white outline thickness of 10 mm and a density of 100%. The inner region has a density of 30%. At the center of the large circle, there are four groups of 12 circles, each with a radius of 3 mm, 5 mm, 7 mm, and 9 mm, respectively, and with equal distances between the circles that are equal to their respective radius sums. The clarity of the dots reflects the resolution of the images.

The parameters for the collimator, as presented in Table 1, have been optimized to keep a balance between spatial efficiency and spatial resolution. On the designed sampling orbit, the proposed slit-slat collimator yields a spatial efficiency of $4.6\times {10}^{-5}$ for phantom, which is higher than that of the traditional parallel-hole collimator $2.3\times {10}^{-5}$ used in SPECT imaging. The design of multiple detectors requires the use of CZT detectors with smaller space constraints, with an intrinsic resolution of approximately 2.4 mm. The combined design of the proposed collimator and the CZT detectors yields a maximum lateral spatial resolution of 4.55 mm. However, it should be noted that the uniformity of lateral spatial resolution among the collimators is not maintained, as the collimators closer to the body exhibit higher lateral spatial resolution. The resolution of this model may further improve as advancements are made in future semiconductor det technology.

**Figure 9:**
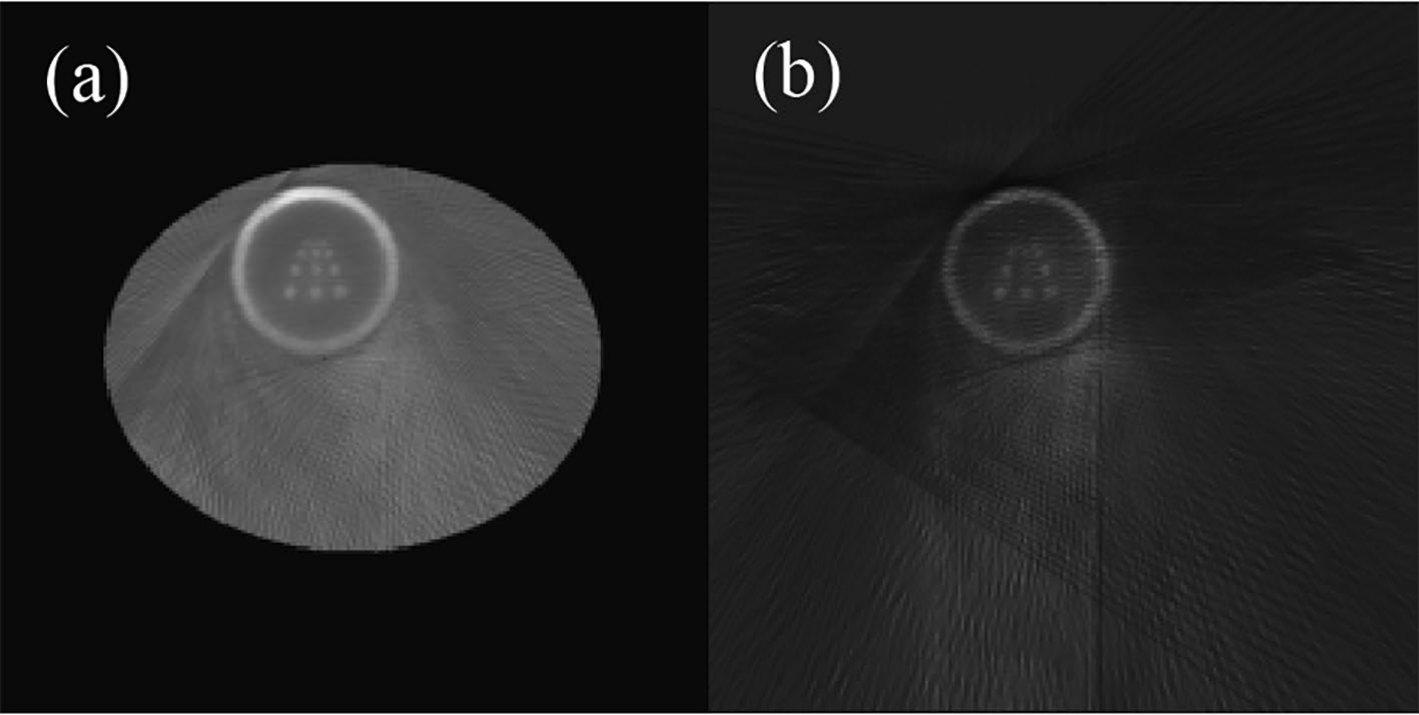
Reconstruction results with (**a**) and without (**b**) incorporating the body contour constraint.

In the ME-SPECT model, the limited 180° sampling of SPECT leads to insufficient information for the complete reconstruction of the entire body. Even the ROI targeted by the slit-slat collimator still exhibits a significant amount of artifacts along the edges of the reconstructed image without depth correction from multiple projections in the lower and lower right part of the circle. However, adding body contour constraints in the iteration algorithm can significantly enhance the image quality, as demonstrated by the comparison between the images with and without body contour constraints in Fig. 9. Both were generated using a standard OSEM algorithm with AC.

**Figure 10:**
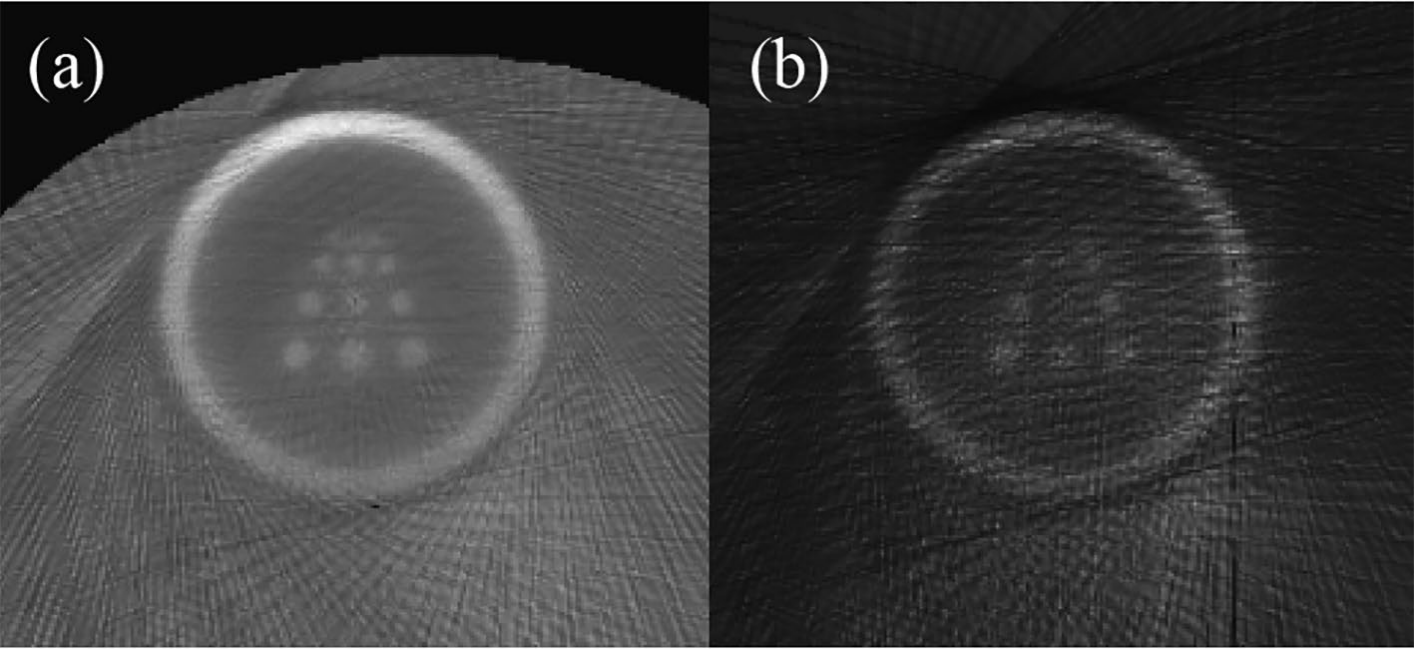
ROI with body contour constraint (**a**) and ROI without body contour constraint (**b**).

In Fig. 9, the improvement in image clarity as a result of the addition of body contour constraints is visually apparent. With such constraints, the reconstruction of the entire body is feasible due to the truncation of SPECT data. Figure 10 provides a comparison of the performance of the two in the ROI. Even within the ROI, Fig. 10b exhibits more artifacts and noise, as well as lower resolution, compared to Fig. 10a.

**Figure 11:**
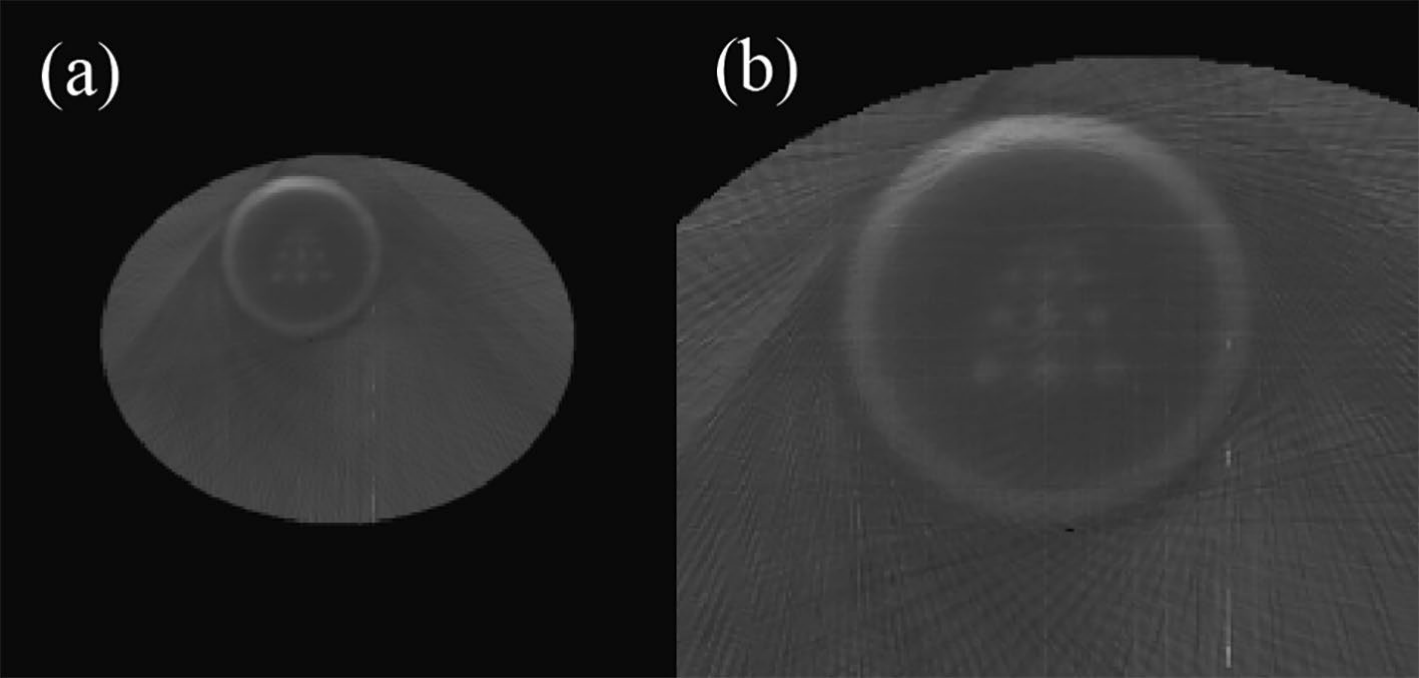
Reconstruction image without AC (**a**) and ROI of reconstruction image without AC (**b**).

The combination of SPECT and CT is critical due to the requirement for AC. In the absence of AC, the reconstructed image is likely to appear blurry and dull. Figure 11 demonstrates the impact of the lack of AC on the image reconstruction process. Compared to Fig. 10a, the image without AC displays severe distortion and interference.

Due to the iterative algorithm's propensity for incorporating constraints, severe data truncation can also serve as prior knowledge to enhance reconstruction quality. As we can see in Fig. 10b, even without any additional constraints, the ROI can still be partially reconstructed to a certain extent, as all detectors are aimed at the ROI. The existence of the CT system allows us to accurately distinguish the ROI area from the non-ROI area. At the end of each iteration, the non-ROI is substituted with the initial image of the body contour. This results in an image that has only reconstructed the ROI. This work is carried out in steps 5 to 14.

**Figure 12:**
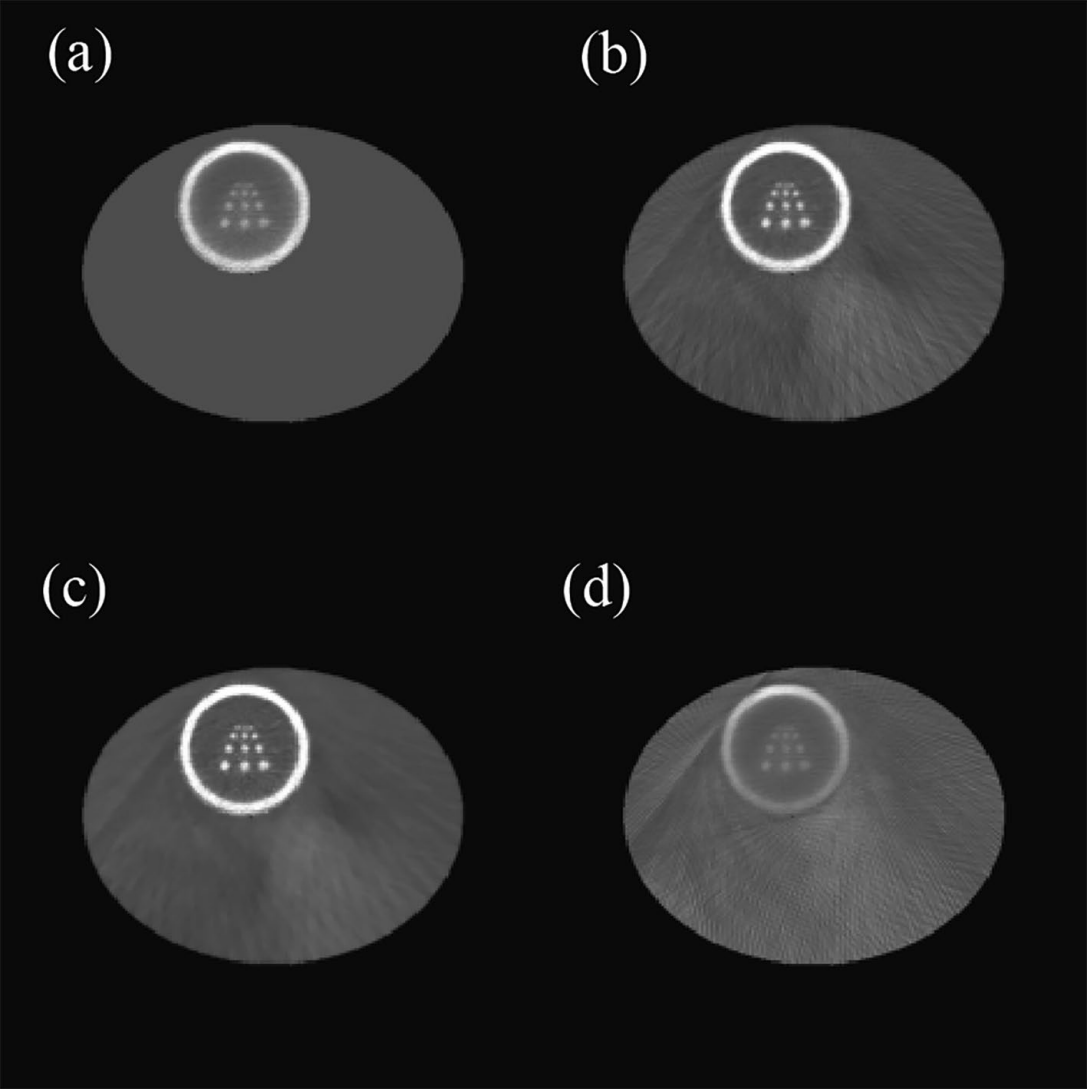
Comparison of the reconstruction results during the image reconstruction process with the standard OSEM algorithm.

The image that only reconstructed the ROI region is used as the initial image for reconstruction, although it is still insufficient to support the recovery of the liver region. It can be seen that the liver region has different densities of a blur. Compared to the standard OSEM algorithm, using the reconstruction results of the liver region more closely aligned with the phantom will improve the reconstruction quality of the heart region. The differences between the two algorithms can be seen in Fig. 12. To evaluate the performance of the proposed algorithm scientifically, we used three commonly used reconstruction quality metrics to assess the quality of the reconstructed results^12^.

1. Normalized mean square distance criterion (NMSD):

10 $$\begin{array}{c}NMSD={\left[\frac{\sum_{i}^{M}\sum_{j}^{N}{\left({t}_{i,j}-{r}_{i,j}\right)}^{2}}{\sum_{i}^{M}\sum_{j}^{N}{\left({t}_{i,j}-{t}_{avg}\right)}^{2}}\right]}^\frac{1}{2}\end{array}$$

2. Normalized mean absolute distance criterion (NAAD):

11 $$\begin{array}{c}NAAD=\frac{\sum_{i}^{M}\sum_{j}^{N}\left|{t}_{i,j}-{r}_{i,j}\right|}{\sum_{i}^{M}\sum_{j}^{N}{|t}_{i,j}|}\end{array}$$

3. Worst case criterion distance (WCCD):

12 $$\begin{array}{c} WCCD=\underset{\begin{array}{c}1<i\le \frac{M}{2}\\ 1<j\le \frac{N}{2}\end{array}}{\mathrm{max}}\left|{T}_{ij}-{R}_{ij}\right|\\ { T}_{ij}=\frac{1}{4}\left({t}_{2i,2j}+{t}_{2i+\mathrm{1,2}j}+{t}_{2i,2j+1}+{t}_{2i+\mathrm{2,2}j+}\right)\\ { R}_{ij}=\frac{1}{4}\left({r}_{2i,2j}+{r}_{2i+\mathrm{1,2}j}+{r}_{2i,2j+1}+{r}_{2i+\mathrm{2,2}j+}\right)\end{array}$$

In the above equation, t is the original image, and r is the reconstructed image. I and j denote the image indices, with the image size being *M* × *N*. ${t}_{avg}$ represents the average value of all pixels in the original image. Among the above indicators, NMSD represents the degree of deviation between the reconstructed image and the original image. NAAD represents the absolute deviation between the reconstructed image and the original image. WCCD represents the maximum error magnitude between the reconstructed image and the original image. The results of the proposed algorithm and the standard OSEM algorithm for various indicators in the ROI are shown in Table 2.

**Table 2:** Comparison of deviation in reconstruction process between proposed algorithm and standard OSEM algorithm.

	Figure 11a	Figure 11b	Figure 11c	Figure 11d
NMSD	0.313	0.227	0.210	0.435
NAAD	0.280	0.172	0.153	0.346
WCCD	0.545	0.438	0.390	0.601

The reconstruction precision of the liver part was improved by using the ROI reconstruction results as prior knowledge. As shown in Fig. 12b, although the liver part was still not reconstructed, a blurred shape was presented compared to the OSEM algorithm. The data in Table 2, combined with Fig. 12c, shows that adding median filtering in the iteration process improved the reconstruction quality of the ROI.

**Figure 13:**
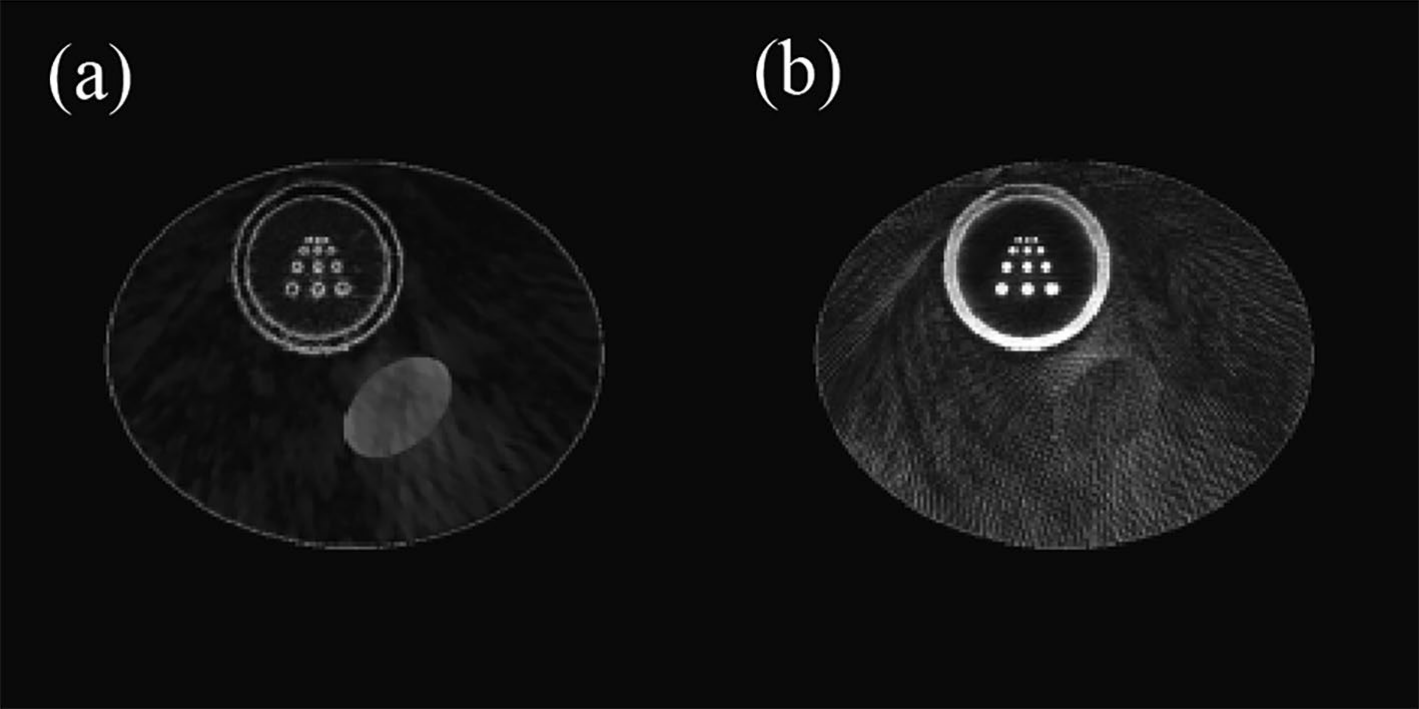
Residual images of the proposed algorithm (**a**) and the standard OSEM algorithm (**b**).

Figure 12 shows the reconstruction results at each stage of the proposed algorithm, and the improvement compared to the standard OSEM algorithm is quite clear. The pseudo-edge around the ROI has been reduced by comparing Fig. 12c,d. In Fig. 12d, there is a blurred edge in the lower right corner of the heart due to insufficient scan sampling, which is much more explicit in Fig. 12c. A more intuitive comparison can be seen in the residual image shown in Fig. 13.

The residual image, which is the absolute difference between the reconstructed image and the original image, is shown in Fig. 13. As can be seen from Fig. 13, the proposed algorithm is almost consistent with the original image in the ROI, and only the outline of the edge region can be seen. This is a significant improvement compared to the standard OSEM algorithm. In addition, as shown in Fig. 13a, the points at 9 mm, 7 mm, and 5 mm are also hollow, and only the outline of the edge can be seen. This indicates that the proposed algorithm has achieved a resolution of 5 mm compared to the various points in Fig. 13b, which can also be seen in the original image. However, there is still a considerable difference in density.

## Discussion

The simulation results demonstrate the superior performance of the proposed ME-SPECT system in SPECT imaging compared to traditional SPECT. ME-SPECT offers several advantages, including faster sampling speed with parallel data acquisition using eight detectors, shorter sampling time with the assistance of CT data, and higher resolution and spatial efficiency in the ROI despite abandoning whole-body scanning. The ME-SPECT system is comparable in size to small myocardial SPECT systems and integrates CT, providing body contour constraints, ROI information, and attenuation mapping matrices for improved reconstruction quality. As shown in Table 2, the proposed algorithm using CT information as prior knowledge exhibits a minor deviation from the original phantom than the standard OSEM algorithm and achieves spatial resolution close to the maximum value achievable with the collimator design. The combination of CT and SPECT in the ME-SPECT system structure offers further development opportunities.

## Conclusion

In conclusion, we present a novel multi-detector parallel sampling ME-SPECT system that effectively improves the system's sensitivity, spatial resolution, and imaging speed. The ME-SPECT system exhibits a sensitivity close to two times that of traditional dual-head SPECT in regions near the heart. The integrated CT system provides SPECT heart position information for adjusting the direction and FOV of the slit-slat collimator and accurate attenuation mapping matrices. We proposed an algorithm that grounds in reciprocal prior knowledge of regions of interest (ROI) and non-ROI, effectively suppressing distortion and artifacts by utilizing the higher spatial resolution provided by multiple detector FOVs. Simulation experiments demonstrate that incorporating this method and providing constraints significantly improves imaging quality. Future work will explore the temporal resolution enhancement in myocardial SPECT/CT parallel sampling.

## Data Availability

All data and codes included in this study are available upon request by contact with the corresponding author.
